# Mitigating background caused by extraneous scattering in small-angle neutron scattering instrument design

**DOI:** 10.1107/S1600576721001084

**Published:** 2021-03-03

**Authors:** John George Barker, Jeremy C. Cook, Jean Philippe Chabot, Steven R. Kline, Zhenhuan Zhang, Cedric Gagnon

**Affiliations:** a NIST Center for Neutron Research, National Institute of Standards and Technology, 100 Bureau Drive, Stop 6102, Gaithersburg, MD 20899, USA

**Keywords:** extraneous scattering, albedo, background, small-angle neutron scattering, SANS

## Abstract

Measurements, calculations and design ideas to mitigate background caused by extraneous scattering in small-angle neutron scattering (SANS) instruments are presented. Scattering includes processes such as incoherent scattering, inelastic scattering and Bragg diffraction. Three primary sources of this type of background are investigated: the beam stop located in front of the detector, the inside lining of the detector vessel and the environment surrounding the sample. SANS measurements were made where materials with different albedos were placed in all three locations. Additional measurements of the angle-dependent scattering over the angular range of 0.7π–0.95π rad were completed on 16 different shielding materials at five wavelengths. The data were extrapolated to cover scattering angles from π/2 to π rad in order to estimate the materials’ albedos. Modifications to existing SANS instruments and sample environments to mitigate extraneous scattering from surfaces are discussed.

## Abstract 2

Measurements and methods of mitigation for small-angle neutron scattering instrument background caused by extraneous scattering from surfaces are presented.

## 1. Introduction   

**Figure 1:**
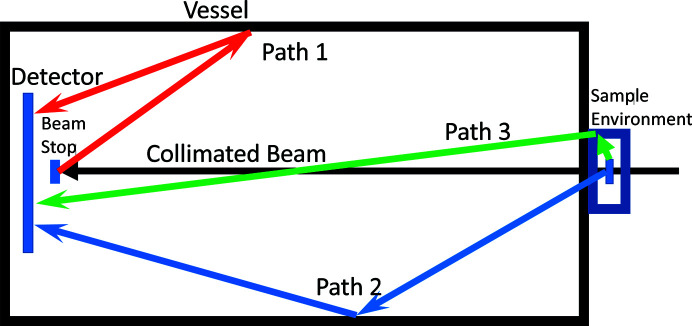
A sketch showing three possible paths whereby scattering can produce background in SANS experiments. A 2D detector is placed inside a vacuum vessel. A sample inside an environment-control apparatus is placed upstream of the vessel. A collimated beam (black line) passes through the sample into the vessel and strikes a beam stop just in front of the detector. Path 1 (red line) represents reflective scattering, first from the beam stop and secondly from the lining of the detector vessel. Path 2 (blue line) represents scattering first from the sample followed by reflective scattering from the detector vessel lining. Path 3 (green line) represents scattering from the sample followed by scattering from the sample environment.

Most current small-angle neutron scattering (SANS) instruments of pinhole aperture design are laid out similarly to the original design of the D11 instrument at the Institut Laue–Langevin (ILL) in the 1970s (Schmatz *et al.*, 1974 ▸), shown schematically in Fig. 1 ▸. A collimated beam impinges upon a sample. A vacuum vessel houses a two-dimensional (2D) detector on rails to allow variation of the sample-to-detector distance and consequently the angular range of neutrons to be collected. The direct beam is blocked by a beam stop just before the detector. The NIST Center for Neutron Research (NCNR) has four pinhole-type SANS instruments. Three of the vessels are lined on the inside with neutron-absorbing cadmium sheet. Due to requirements of lower cost, health and environment considerations (D’Mellow *et al.*, 2007 ▸) and lower γ-ray dose, sheets of flexible elastomer containing neutron-absorbing boron (B-Flex) from two different suppliers were used to line the most recently constructed ‘very SANS’ (vSANS) detector vessel (Barker, 2020 ▸). The proprietary B-Flex materials are a blend of a natural boron material such as boron carbide powder with an elastomeric matrix. The lining serves two purposes: to absorb neutrons from the exterior entering the vessel and to minimize the scattering incident on the inside surface. The B-Flex flexible sheet materials conform easily to the shape of the vSANS vessel and are attached with a silicone adhesive. This work will show that the cadmium sheet has less scattering than the B-Flex, thereby providing a lower instrument background. The rail supports and electrical cable carriers are currently unshielded, providing additional surface areas with higher extraneous scattering inside all the vessels.

The neutron albedo of a material is the fraction of all incident neutrons that reflect from its surface (Price *et al.*, 1957 ▸; Shultis & Faw, 1996 ▸). Any such scattered neutrons that reach the detector from materials other than the sample are a source of background. In a SANS experiment, measurements are run on the sample, the empty cell and a cell filled with an absorber such as Cd to block the beam. The empty-cell and beam-blocked runs are used to subtract background scattering as described by Brûlet *et al.* (2007 ▸). In Fig. 1 ▸, the red ray shows schematically how scattering from the intense direct beam incident on the beam stop, followed by reflective scattering from the inside the vessel, can reach the detector in a two-step process. This source of background is typically subtracted via the empty-beam, *i.e.* empty-sample-cell, background measurement during data reduction. However, for weakly scattering samples the measurement error is increased significantly if the scattered background is large in comparison to the sample scattering. The blue ray in Fig. 1 ▸ shows how the weaker larger-angle scattering from the sample can reflect from the vessel lining to the detector in a one-step process. This second background remains after data correction. Typically, it is observed as an enhancement of the flat background component from incoherent or inelastic scattering. The third source of scattered background is linked to the sample holder and nearby components of ancillary equipment. A typical sample produces an intense background at large scattering angles from incoherent scattering, inelastic scattering or wide-angle diffraction. This large-angle scattering (green ray) interacts with materials in the sample environment surrounding the sample and can redirect the neutrons into the detector. This third background source is again not removed by empty-cell subtraction. All three sources of background appear as nearly flat scattering on the detector.

This paper describes a series of measurements of all three sources of extraneous scattering background. The measurements were made on the 30 m SANS instrument (Glinka *et al.*, 1998 ▸), now located on neutron guide NG-Bu, and on the newly commissioned vSANS instrument on guide NG-3 which has three separate detector carriages on rails (Barker, 2020 ▸). Each background source is described in more detail in a separate section below. To be able to estimate the amount of scattering background, we need first to estimate the angle dependence of the scattering with a range of incident wavelengths and a range of materials used in instrument construction. Recently, scattering measurements were made on common shielding materials using thermal neutrons (0.1 ≤ λ ≤ 2.5 Å) by Stone *et al.* (2019 ▸). The current measurements extend such data to cover cold neutrons of wavelengths 4.5 ≤ λ ≤ 15 Å. The measurements were made on an absolute scale over a large scattering angle range from 0.7π to 0.95π rad. The experimental setup has the incident and scattered beams in a vacuum of 0.1 Pa air pressure, producing very low background and thus allowing the measurement of very low albedo materials such as gadolinium foil. Table 1 ▸ lists the 16 materials used in this study. Boron carbide is extensively used at other facilities (Dewhurst *et al.*, 2016 ▸) but it is an extremely hard material, making fabrication difficult. It was unavailable for this study but we include it in Table 1 ▸ for the purpose of comparison. By extrapolating the data to include scattering angles θ from π/2 to π rad, the fraction of the beam that is reflected, which is called the albedo *F*, is estimated. Finally, we discuss design methods to mitigate all three background sources.

**Figure 2:**
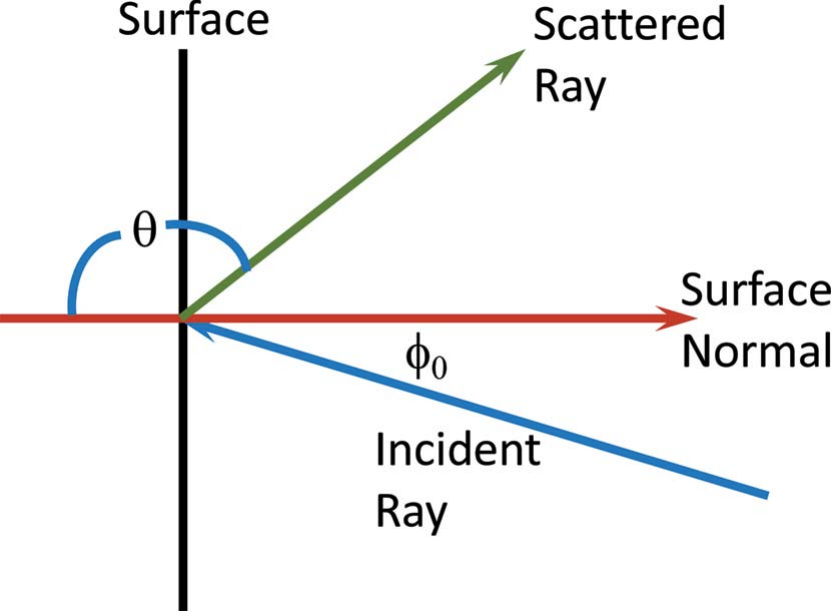
Diagram showing the definition of angles for scattering. Both the scattering angle θ of the scattered ray and the angle of incidence ϕ_0_ of the incident ray are defined with respect to the surface normal. All measurements in this paper have ϕ_0_ = 0.

Angle-dependent scattering *S*(θ) as used in this paper is closely related to the absolute scattering cross sections *I*(*q*) used in SANS, where *q* is the momentum transfer [*q* = (4π/λ)sin(θ/2), θ is the scattering angle and λ is the wavelength of the incident radiation]. Whereas *I*(*q*) is typically reported in units of cm^−1^ sr^−1^, here the scattering *S*(θ) is defined in units of sr^−1^. The reflective scattering angle range θ is π/2 ≤ θ ≤ π rad, where the neutron exits the front surface of the material, *i.e.* the surface upon which the neutron beam is incident. The reflected scattering saturates for an infinitely thick sample. Ideally, both the lateral extent and the thickness of the test sample are infinite, but lengths extending to optical thickness τ > 3 are generally adequate for the measured scattering to be nearly equivalent to the infinite slab case; τ ≡ *t*Σ_tot_, where *t* is the sample thickness and Σ_tot_ is the macroscopic total cross section. Fig. 2 ▸ shows a schematic of the defined angles with respect to a flat surface. The angle between the incident neutron trajectory and the surface normal is defined as ϕ_0_. The angle of the scattering from the surface normal is defined as θ. The scattering fraction as distributed over solid angle is
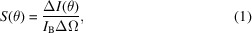
where *I*_B_ is the incident beam current and Δ*I*(θ) is the scattered intensity measured at an angle θ with a detector element having solid angle ΔΩ. Note that for such thick samples multiple scattering interactions are very likely. The scattering *S*(θ) will thus also depend upon the incident orientation of the beam ϕ_0_. The albedo *F* is calculated by integrating over solid angle in the reflective scattering direction,
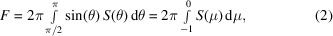
where μ = cos(θ).

The albedo *F* is constrained by the limits 0 ≤ *F* ≤ 1, with the lower limit corresponding to none and the upper limit to all of the neutrons being reflectively scattered. In extrapolating scattering data to small μ, we use the approximation
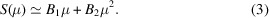
Note the above expression produces *S*(θ) = 0 at θ = π/2 rad. In this direction the scattering is attenuated by the ideally infinite lateral extent of the sample. Equation (3) agrees well with semi-analytical calculations made by Chandrasekhar (1960 ▸) and Monte Carlo simulations by Barker & Mildner (2015 ▸) for isotropic incoherent scattering. The direction of single Bragg scattering events is dependent upon the incident direction and not the surface normal. But in the limit as ϕ_0_ → 0, the incident direction and the surface normal coincide so that the scattering distribution from Bragg scattering from polycrystalline materials with uniform texture should also be azimuthally isotropic. For normal incidence of the beam to the sample where ϕ_0_ = 0 and using Bragg’s law λ = 2*d*sin(θ/2), Bragg scattering with angular range constraint π/2 ≤ θ ≤ π occurs with the wavelength λ constrained to the crystalline *d* spacing as λ/2 ≤ *d* ≤ λ/2^1/2^.

To minimize background on the detector, materials of low albedo should be used as beam stops, as lining for the detector vessel and in critical areas of the sample environment. Note that *F*(λ) ≤ ω_0_ = [Σ_tot_(λ) − Σ_abs_(λ)]/Σ_tot_(λ), where the subscripts tot and abs represent the total and absorption macroscopic cross sections of the material, respectively. Our interest is in cold neutrons undergoing thermal atomic interactions resulting in scattered wavelengths well within the range 0.01 < λ < 15 Å. Tabulations of the wavelength dependence of the total cross section Σ_tot_ of different elements (Goldberg & Mughabghab, 1966 ▸) and water (Mattes & Keinert, 2005 ▸) show that the absorption cross section depends linearly on wavelength, Σ_abs_(λ) = *A*λ, unless near a resonance which occurs for cadmium (Cd) or gadolinium (Gd). The remaining scattering part of the total cross section shows a more complicated dependence. At short wavelengths 0.01 < λ < 0.1 Å, the scattering cross section is typically constant with Σ_scat_(λ) = Σ_tot_(λ) − Σ_abs_(λ) ≃ Σ_inc_ + Σ_coh_, where the subscripts inc and coh represent incoherent and coherent scattering cross sections, respectively. At these wavelengths the neutron velocity *v*_n_ is much larger than the average atomic velocity *v*_a_ in the material, *v*_n_ >> *v*_a_. At intermediate wavelengths 0.1 < λ < 10 Å, the remainder cross section has complicated elastic and inelastic contributions from Bragg and phonon scattering, since the neutron and atomic velocities are comparable and these wavelengths enter the range of atomic spacings. At very large wavelengths, where the atomic velocity greatly exceeds the neutron velocity, *v*_a_ >> *v*_n_, and Bragg scattering is no longer possible, the scattering cross section simplifies to Σ_scat_(λ) = Σ_tot_(λ) − Σ_abs_(λ) ≃ [Σ_inc_ + Σ_coh_] *g*λ, where *g* is a constant dependent upon the inelastic scattering cross section and is material and temperature dependent. *g* is typically smaller for higher melting point materials due to the tighter atomic bonding (Steyerl, 1977 ▸; Freund, 1983 ▸; Barker & Mildner, 2015 ▸).

Once a neutron scatters, it must again transit the sample to escape. The path length for scattered neutrons is thus enhanced. The path length is further enhanced by multiple scattering events. The longer path length allows for further absorption of the scattered neutrons. Thus, the fraction of neutrons in the albedo must be *F* ≤ Σ_scat_/Σ_tot_. The predicted limiting behavior of the albedo is then 
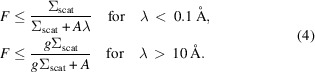



At long wavelengths beyond the Bragg cutoff (λ > 2*d*_m_, where *d*_m_ is the maximum crystalline *d* spacing), the albedo *F* is expected to be at its lowest and to be relatively constant due to the dominant inelastic scattering process, as well as being proportional to wavelength. At short wavelengths below the Bragg cutoff, the albedo *F* is expected to increase as the scattering saturates and the absorption dissipates. The lowest-albedo materials should have the lowest scattering to absorption coefficient ratio Σ_scat_/*A*. Of all the shielding materials listed in Table 1 ▸, ^10^B-enriched boron carbide (^10^B_4_C) has the lowest ratio. Cd and Gd are the next lowest but have higher ratios by factors of three and four, respectively.

## 2. Albedo measurements   

To estimate background in SANS instruments from the extraneous scattering shown in Fig. 1 ▸, we need to estimate on an absolute scale the scattering *S*(θ) and albedo *F* for mater­ials used in instrument construction. In principle the information can be obtained by knowing the isotopic composition of the materials and their neutron cross sections, and modeling the scattering using Monte Carlo neutron transport programs such as *MCNP* (Briesmeister, 1997 ▸) or *McStas* (Willendrup *et al.*, 2004 ▸). Programs exist to calculate the scattering kernels (Boin, 2012 ▸), but properly incorporating inelastic and Bragg scattering is difficult. The grain-size distribution, preferred grain orientation (texture) and dislocation structure all have an impact on the calculation of the Bragg scattering component. Direct experimental measurements of the albedo *F* and scattering *S*(θ) on such materials can validate kernel calculations. Stone *et al.* (2019 ▸) recently measured scattering on an arbitrary scale for a number of relevant materials at thermal neutron energies. Here we extend such measurements on an absolute scale to the cold neutron energies used in SANS measurements. The large scattering angular range of the current measurements also allowed estimates of the albedo *F* of the materials.

**Figure 3:**
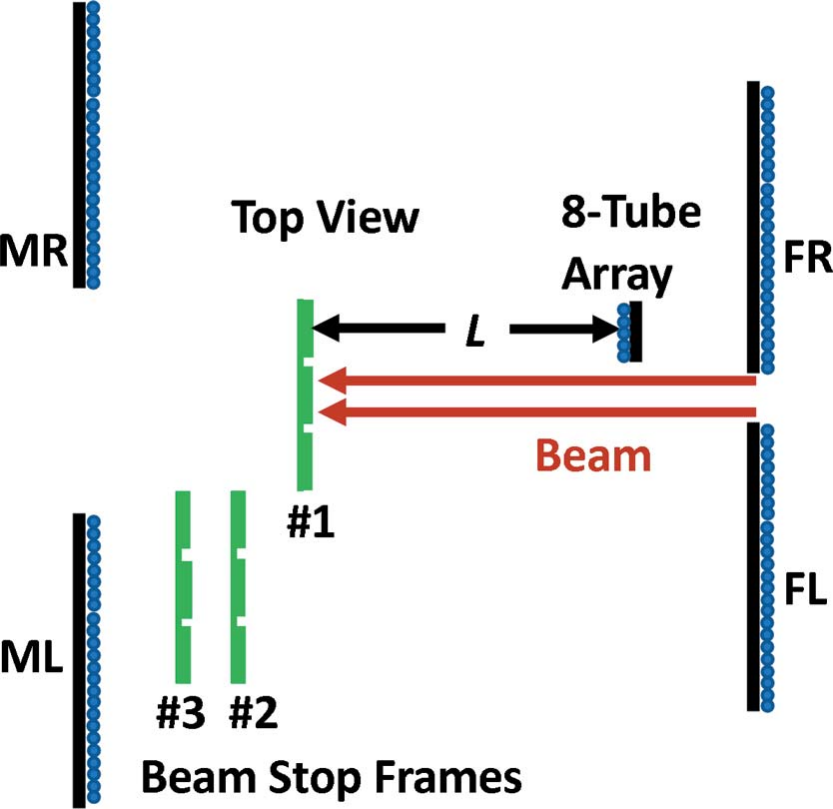
A top-view schematic showing the experimental setup for measuring the albedo *F* and scattering *S*(θ) in Section 2. The three beam-stop support frames are shown in green, with frame #1 in the beam (red). Six different samples are mounted on each frame. The tube detectors are shown in blue, with ^10^B-Al shielding in black. The 48-tube vSANS detector panels are labeled FL, FR, ML and MR, where the panel label names use L and R to represent the left and right panels, respectively, and F and M to represent the front and middle detector carriages, respectively. The eight-tube array used for the albedo measurements is shown facing the beam stops. The beam is offset 50 mm from the side of the eight-tube array. The three beam-stop frames are at distances *L* = 0.36 (as shown), 0.44 and 0.49 m from the eight-tube array.

Our measurements were made on the newly commissioned vSANS instrument at the NCNR. Fig. 3 ▸ shows a schematic diagram of the scattering measurement equipment arrangement. Three thin (1.5 mm thick) 200 × 200 mm aluminium sheets were attached to hollow aluminium support tubes attached to motorized slides located on the front of the middle carriage, allowing translation in both the horizontal and vertical directions. The four detector panels on the middle carriage were all translated out to make a large 22 cm wide by 24 cm tall central opening that allows the direct beam to pass unimpeded. Up to six different beam-stop materials were attached to each frame. For each measurement, the frame was translated horizontally and vertically to place one material in the beam at a time. Eight ^3^He tube detectors were placed upstream and facing the beam stops to observe the backscattered neutrons. The upstream side of this tube array was shielded. This detector array was placed on the back of the front (most upstream) carriage facing the middle carriage. The four detector panels on the front carriage were adjusted to allow a 5 cm wide by 21 cm tall opening which allows the 5 cm diameter beam to pass but blocks much of the background produced by the 4 cm long air path and 0.6 cm thick silicon and 0.3 cm thick sapphire windows located 2 m upstream. Thus, the front carriage detectors block most of the background scattering from the sample area from reaching the back-facing detector array. The front and middle carriages were positioned as close as possible together to maximize the scattering angle range collected. The distances from the beam-stop frames 1, 2 and 3 to the back-facing detector array were 0.36, 0.44 and 0.49 m, respectively. From these distances the thickness of the individual beam stop was subtracted when determining scattering angles. The beam center passes 50 mm to the side of the edge of the detector array. The eight-tube detection array covered an area of 67 mm wide by 1040 mm tall. The angular coverage was approximately 0.7π–0.95π rad for frame-1 beam stops, with the angular limits for the other two frames altered by the longer distance.

Measurements were made at five wavelengths, λ = 4.5, 6, 8, 12 and 15 Å. The wavelength was selected using a helical velocity selector having an FWHM resolution of Δλ/λ = 12.5%. The beam current incident on the beam stops was determined by directly measuring the beam, attenuated by a calibrated poly(methyl methacrylate) (PMMA) beam attenuator, with a middle carriage tube detector panel. All 16 of the studied materials were placed to stop the beam and the scattering was measured at each wavelength. An observed count rate of 1.1 s^−1^ uniform background on the eight-tube array measured with the instrument shutter closed (dark current) is attributed to neutron background from neighboring instruments and was subtracted during data reduction. The reactor-off background on the array is 0.02 s^−1^. All the data in this section’s tables and figures have the dark-current background subtracted, but this correction was significant only for the low-albedo materials such as Gd. Table 2 ▸ shows the beam current and the count rate measured on the rear-facing detector array with a low-albedo Cd beam stop. The data were radially averaged with the same software as used for SANS data reduction (Kline, 2006 ▸) but with some parameters altered to reflect the new geometry. The output scattering angle θ′ was reset to θ = π − θ′ based upon the rear-facing geometry. The horizontal tube spacing was 8.4 mm. The charge division encoded 128 positions along the tube axis at 8.14 mm spacing.

**Figure 4:**
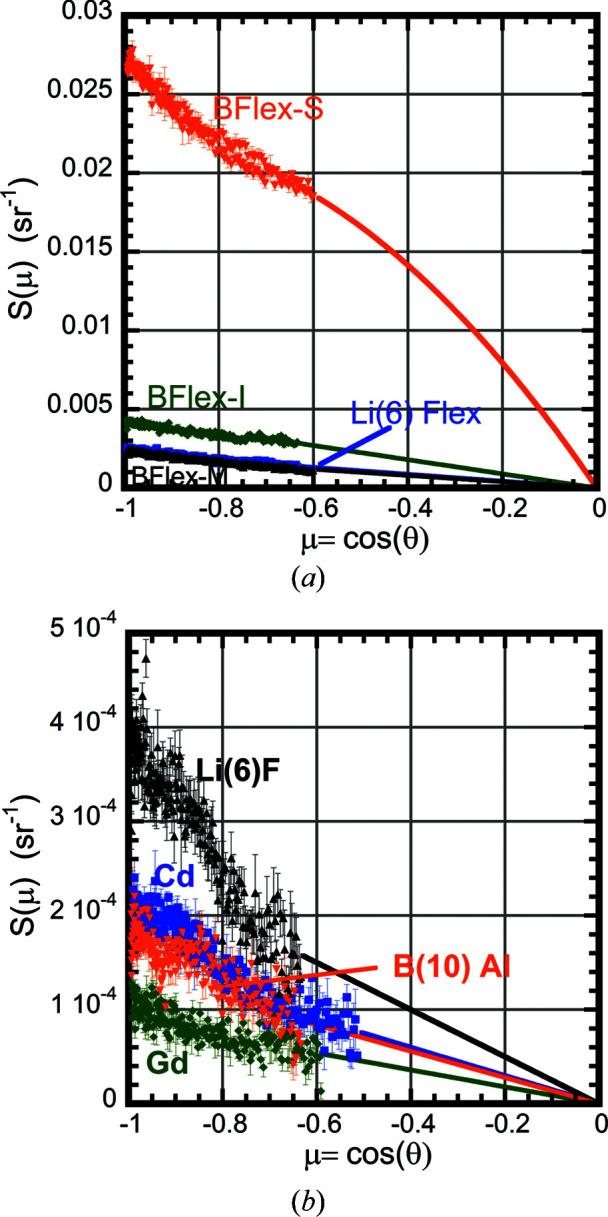
Plots of the scattering fraction *S*(μ) for several materials at λ = 4.5 Å. The symbols represent experimental data and the solid lines are extrapolated fits according to equation (3). The error bars in all figures represent one standard deviation in uncertainty. (*a*) Plots for the four flexible elastomer samples: ^6^Li-Flex (blue), B-Flex-I (green), B-Flex-M (black) and B-Flex-S (orange). (*b*) Plots for the four strongly absorbing materials: Cd (blue), Gd (green), ^6^LiF (black) and ^10^B-Al (orange).

Table 3 ▸ lists the measured albedo *F* for all 16 materials. Transmission measurements were used to estimate the optical thickness τ for the thinner samples. For the thicker samples we used the absorption cross sections from Table 1 ▸ to estimate τ. First, the integration was done only over the angular range of the data acquired to produce a partial albedo Δ*F*. The data were then linearly fitted to μ versus *S*(θ)/μ to extrapolate the data to μ = 0. In two cases [poly(tetra­fluoro­ethyl­ene) (PTFE) at λ = 8 Å and BN at λ = 6 Å], the extrapolation also included the calculation of Bragg scattering from a single reflection. The solid lines in Figs. 4 ▸ and 5 ▸ indicate that the fits provide a smooth extrapolation of the data. The extrapolated data were used to obtain the full albedo *F*.

Four of the materials tested were made from flexible elastomer sheets impregnated with absorbing elements. The hydrogen within the elastomeric phase is the dominant source of albedo for these materials. ^6^Li-Flex is a material loaded with ^6^LiF inside an elastomeric matrix. The ^6^Li isotope does not produce γ-rays. The material is often used to block intense neutron beams while minimizing γ production, thus minimizing the amount of biological γ-ray shielding needed to lower dose levels to a safe range. Three natural boron-loaded elastomeric B-Flex materials from different vendors were tested. ^10^B capture produces predominantly 478 keV γ-rays from the (*n*, α) channel. The γ-ray dose rate per capture from ^10^B is one order of magnitude lower than that of most other shielding materials, thus also minimizing expensive biological shielding. Each B-Flex has similar boron-loading densities, based upon the measured absorption cross sections used to determine the optical thicknesses τ given in Table 3 ▸. Fig. 4 ▸(*a*) shows the scattering for all four elastomeric materials at λ = 4.5 Å. The B-Flex type S has a higher scattering background by a factor of five to ten than the other two B-Flex materials tested. This is likely to be due to a lower boron density near the surface allowing enhanced hydrogen scattering. Thus, neutrons are more likely to scatter from the elastomeric phase without entering the boron-containing phase. The inside of the vSANS vessel is covered 45% each with the S and I types of B-Flex sheeting, with the remainder being exposed metal, plastic and electrical cabling.

Beam-defining apertures and beam stops are often made using highly absorbing materials to reduce the scattered background. Because they are in the incident neutron beam, often the absorbing isotope chosen is ^6^Li or ^10^B to reduce the γ-ray dose rate. At NIST, the ^6^Li-Flex, ^6^Li-Glass, ^6^LiF, ^10^B-Al, boron nitride (BN), Cd and Gd materials are all used for this purpose. A layered structure is often used for apertures, where the first layer has lower γ production and the back layer is made from either Cd or Gd to have a lower amount of scattering from the inner edge of the aperture. Fig. 4 ▸(*b*) shows the scattering for four highly absorbing materials (Cd, Gd, ^6^LiF and ^10^B-Al) at λ = 4.5 Å. The lowest albedo *F* was measured on Gd, a factor of two to four better than the other materials. At longer wavelengths where Bragg scattering is absent, Gd and ^10^B-Al have comparable albedos. From Table 1 ▸, the Σ_scat_/*A* ratio is lowest for Cd, but all four materials’ ratios are within a factor of two of each other. Boron carbide enriched in ^10^B has a Σ_scat_/*A* ratio a factor of three lower than even Cd, suggesting it may have the lowest albedo *F* of all.

**Figure 5:**
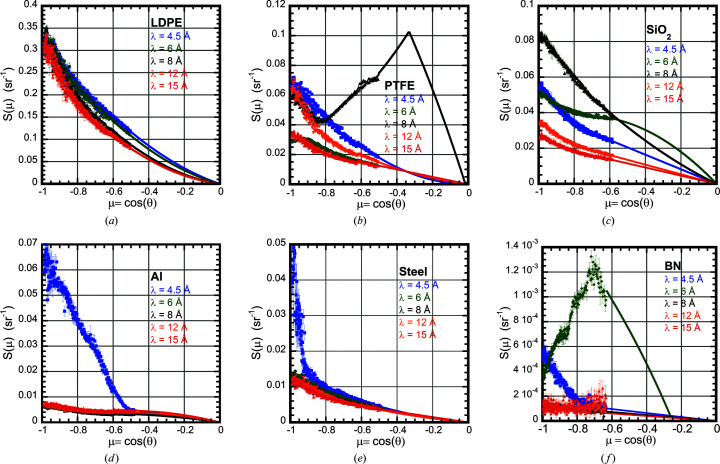
Plots of the scattering fraction *S*(μ) for several other materials at wavelengths 4.5 Å (blue), 6 Å (green), 8 Å (black), 12 Å (orange) and 15 Å (red). (*a*) The LDPE material. (*b*) The semi-crystalline PTFE material. The large increase in scattering near μ = −0.35 at λ = 8 Å is caused by the 100 Bragg reflection. (*c*) The amorphous SiO_2_ material. The broad amorphous diffraction peak enhances the background for λ = 4.5, 6 and 8 Å. (*d*) The thick aluminium 6061 alloy material. The large increase in the low-μ scattering at λ = 4.5 Å is due to strong Bragg scattering from the f.c.c. 111 reflection with *d* = 2.338 Å. (*e*) The low-carbon steel material. Strong Bragg scattering from the b.c.c. 110 reflection with *d* = 2.027 Å is evident at low μ for λ = 4.5 Å. (*f*) The polycrystalline boron nitride (BN) material. Strong Bragg scattering is observed from the hexagonal 100 reflection with *d* = 2.167 Å at λ = 4.5 Å and from the 002 reflection with *d* = 3.309 Å at λ = 6.0 Å.

Fig. 5 ▸(*a*) shows the scattering as a function of wavelength from low-density polyethyl­ene (LDPE). The high hydrogen content makes this material a good reflector, with an albedo *F* of over 60%. The albedo decreases slightly with increasing wavelength, probably due to increased absorption. The PMMA material also had a very high reflectance from its high hydrogen content, with a slightly lower albedo *F* than LDPE, probably due to the thinner sample used. The PTFE semi-crystalline fluoro­carbon mater­ial is expected to have a much lower reflectance due to the removal of hydrogen, but the Bragg scattering from the semi-crystalline phase is quite strong. The 100 Bragg peak from PTFE has a *d* spacing of 4.902 Å, *q* = 1.28 Å^−1^ (Brown *et al.*, 2008 ▸). Fig. 5 ▸(*b*) shows the scattering as a function of wavelength for the PTFE material. Note the strong Bragg scattering from the 100 reflection observed at λ = 8 Å. Following Bragg’s law, 6.93 ≤ λ ≤ 9.8 Å has the 100 peak in the reflective direction; therefore it is not observed in the data at the other wavelengths. The Bragg scattering causes the albedo to spike a factor of three times higher at λ = 8 Å compared with the other measurements. Fig. 5 ▸(*c*) shows the scattering from amorphous SiO_2_. Note the glass structure produces broad scattering from the first scattering peak that enhances the albedo for λ = 6 and 8 Å. Fig. 5 ▸(*d*) shows the scattering from a thick aluminium sample. The Bragg scattering from the face-centered-cubic (f.c.c.) 111 reflection with a *d* spacing of 2.338 Å is dominant at λ = 4.5 Å, producing an albedo *F* a factor of five larger than seen at the longer wavelengths. Fig. 5 ▸(*e*) shows the albedo scattering from steel. The largest *d* spacing from steel, which has a body-centered-cubic (b.c.c.) crystal structure, is the 110 reflection with *d* = 2.027 Å. Due to the wavelength spread of 12.5%, the wavelength range λ = 4.5 Å extends over the range 3.94 ≤ λ ≤ 5.06 Å and the Bragg cutoff is for λ > 2*d* = 4.054 Å. Thus, the steep upturn of the λ = 4.5 Å data near μ = −1 is from the shortest wavelengths that scatter with θ ≃ π. Fig. 5 ▸(*f*) shows the scattering from boron nitride with a hexagonal crystal structure. Strong Bragg scattering is observed at λ = 4.5 Å from the 100 reflection with *d* = 2.167 Å and at λ = 6.0 Å from the 002 reflection with *d* = 3.309 Å. Bragg scattering from smaller *d*-spacing reflections enhances the albedo for wavelengths shorter than those measured here.

For cold neutrons of wavelength λ > 2*d*_m_ Bragg scattering is absent and an inelastic scattering component with cross section proportional to wavelength is expected to dominate at the longest wavelengths. In the absence of Bragg scattering the lowest albedo *F* measured was from ^10^B-Al and Gd, but the metals tested showed very strong Bragg scattering that would increase albedo at shorter wavelengths. Smaller grain sizes combined with high dislocation densities and substructures that increase the mosaic spread all contribute to larger Bragg attenuation in polycrystalline metals. Heat treatment of alloys for several hours at temperatures above half the melting point often produces grain growth and the removal of dislocations by grain-boundary migration. Due to the general lack of dislocations in ceramic materials, the mosaic spread tends to be much lower. It is expected that ^10^B_4_C in polycrystalline form will have a very low albedo even at short wavelengths, due to its small crystalline mosaicity. In summary, the lowest-albedo materials in Table 3 ▸ were found to have the lowest total scattering to absorption constant ratio Σ_scat_/*A*. Materials containing hydrogen had the highest albedos.

## 3. Beam-stop scattering background   

The reflective scattering from the beam stop is incident upon the inside lining of the detector vessel. Reflective scattering from the vessel lining can produce a background component on the 2D detector, indicated by the red rays in Fig. 1 ▸. The ideal beam-stop material will have the lowest albedo possible. But to minimize the γ-ray dose rate produced by the strong direct beam, ^6^Li and ^10^B are preferred over Gd and Cd. The ideal lining material will also have the lowest albedo but does not need to have low γ-ray production because of the low neutron fluxes being shielded. The lining material also needs to have a low cost to cover a large area, and be structurally tough to avoid forming dust that could be a health hazard or damage vacuum pumps. In the following measurements, several different candidate beam-stop materials were chosen to show their performance in reducing background. The tests were repeated with the Cd lining covered by thin PMMA sheets to increase the albedo and thus mimic the elastomeric B-flex materials containing hydrogen.

Measurements were made on the NGB 30 m SANS instrument with the detector at *L* = 13 m distance from the sample, λ = 5 Å and an incident beam current of 1.8 × 10^6^ s^−1^. The air pressure in the vessel was 2.4 Pa. The beam path in the sample area was evacuated to a pressure of 20 Pa to minimize air-scattering background. A dark background current of 1.22 s^−1^ was subtracted to remove detector electronic noise and fast neutron background from other instruments. The detector count rate was collected over the entire active detector area of 0.65 × 0.65 m. To remove the effect of the parasitic halo around the beam stop, a 0.25 × 0.25 m central area was excluded. The scattering from the 0.6 cm thick silicon and 0.3 cm thick sapphire windows surrounding the sample area was estimated to be 0.41 s^−1^ by extrapolating the distance dependence of the detector count rate to *L* = ∞. Measurements were made with beam stops made from the following materials: Cd, ^6^Li-Flex, B-Flex-I, and 0.9 mm thick PMMA backed by Cd. Ninety per cent of the vessel interior surface area is covered by 0.5 mm thick Cd sheet with 10% exposed aluminium rail supports. The tests were repeated with 56% of the vessel lining area covered in 0.9 mm thick PMMA by placing 150 cm tall sheets above the rails on the left and right sides along the entire length of the vessel. From transmission measurements the optical thickness of the PMMA sheeting was τ = 0.41. Assuming purely incoherent scattering and using Chandrasekhar’s *XY* functions (Chandrasekhar, 1960 ▸), the reflected fraction was calculated as 0.511 for τ = 0.41. The albedo determined from the reflected fraction multiplied by the fraction scattered is *F* = 0.511[1 − exp(−τ)] = 0.17. The different albedos of the different beam-stop materials determine the strength of the background incident upon the lining, and the different albedos of the different lining materials determine the strength of the rescattering into the detector.

The 25–35 cm separation of the beam stop and detector allows lines of sight between the vessel lining and the part of the detector behind the beam stop. The presence of scattered background from the beam stop over the entire detector area confirms that the former is distributed over large angles with respect to the beam direction. Table 4 ▸ shows the observed background fraction, *k*_m_, of the beam intensity *I*_B_ seen on the detector after correcting for the dark current and window scattering backgrounds. *k*_m_ is found to scale with the measured albedos *F* found in Table 3 ▸. Adding 0.9 mm of PMMA to the front of the Cd beam stop increased *k*_m_ by a factor of 260, whilst the ratio of albedos *F* for 0.9 mm of PMMA to Cd is 310. Covering 56% of the vessel lining area with 0.9 mm thick PMMA increased *k*_m_ by a factor of three to seven, depending upon the beam-stop material. Table 5 ▸ shows the albedo 〈*F*〉 averaged over the area of the vessel at λ = 4.5 Å with and without the PMMA addition, showing that the latter increases 〈*F*〉 by a factor of 8.5.

Lowering the background from the beam stop is achieved by using the lowest-albedo materials possible for both beam stop and detector lining. From Table 1 ▸ ^10^B_4_C, having the lowest Σ_scat_/*A*, is the best choice for the small-area beam stop. However, the large area of coverage for the detector lining requires a cheaper (and more pliable) material. Cadmium sheet is the most economical and is used to line the vessels of three of the SANS instruments at the NCNR. The newest vSANS instrument vessel is lined with two types of B-Flex, roughly 45% each of I and S types, which have been demonstrated in the present work to be inferior to Cd, and therefore we plan to reline the vSANS vessel with Cd. Additional effort will be made to cover many of the currently exposed metal rails and cable carriers in cadmium sheet as well. In contrast to other SANS instruments at the NCNR, the vSANS detector panels can be arranged to produce a central rectangular hole, allowing beam passage without the need for a beam stop before the detector. Consequently, the beam stop can be placed downstream at the end of the vessel, further mitigating beam-stop-dependent background.

## 4. Vessel albedo background   

**Figure 6:**
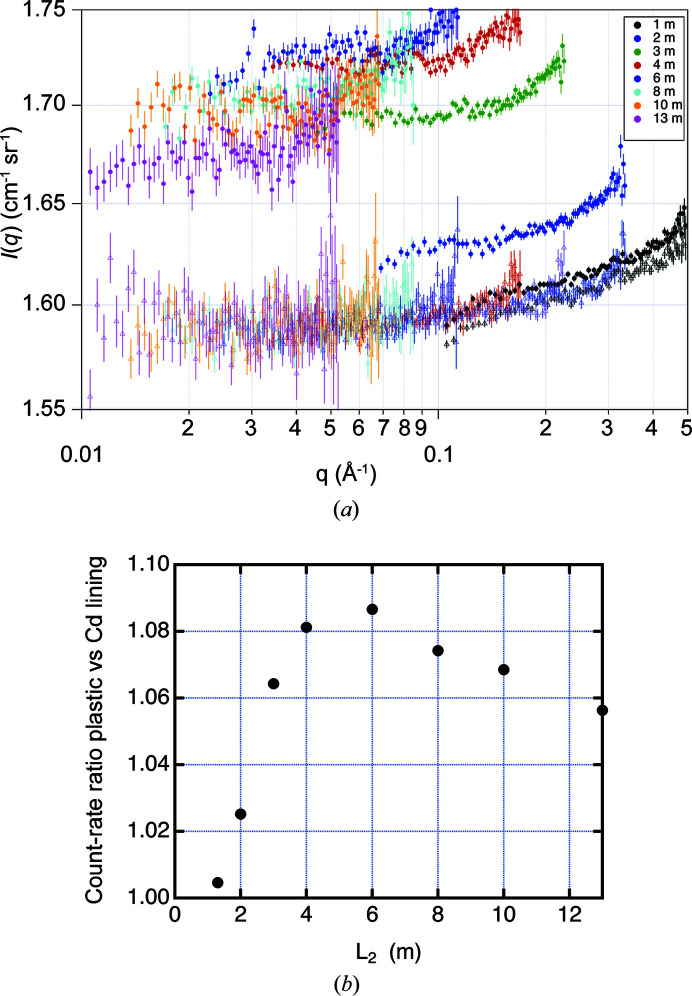
(*a*) Plots of the scattering from the 2 mm thick water sample measured for sample-to-detector distances *L* of 1.3 m (black), 2 m (dark blue), 3 m (green), 4 m (red), 6 m (purple), 8 m (cyan), 10 m (orange) and 13 m (magenta). Background-corrected absolute cross sections from water, as measured with Cd-only detector lining, are shown as open triangles, whereas measurements with the added plastic sheet are shown as filled circles. (*b*). The ratio of the count rate from water observed with plastic and Cd linings as a function of the sample-to-detector distance, *L*. Ratios larger than one indicate additional extraneous scattering from PMMA sheets.

As shown in Fig. 1 ▸, neutrons scattered by the sample can impinge upon the cylindrical vessel lining. Reflective scattering from the vessel lining (blue ray) can then travel to the detector. To test for the existence of this background source, the following experiment was conducted on the NGB 30 m SANS instrument. The measurement was repeated with and without the PMMA plastic sheeting addition to the vessel lining, as described in the previous section. Using an incident neutron wavelength of λ = 6 Å, scattering from a 2 mm thick water sample, an empty cell and a beam-blocked (dark current) cell were all measured at a series of sample-to-detector distances *L* = 1.3, 2, 3, 4, 6, 8, 10 and 13 m. Identical incident-beam collimations were maintained with four guides inserted for the 3 ≤ *L* ≤ 13 m measurements so that the beam current *I*_B_ in the absolute calibration remained unchanged, facilitated by using a large 12.5 cm diameter Cd beam stop. For the *L* = 1.3 and 2 m measurements only one guide was inserted to keep the detector count rate below 5 × 10^4^ s^−1^. This required a separate beam-current measurement for absolute calibration. The scattering cross section *I*(*q*) is plotted in Fig. 6 ▸(*a*) for all *L* with and without the plastic sheeting. It is observed that the ‘without plastic sheeting’ data sets overlap fairly well within statistical error for each *L* at all *q*, whereas the ‘with plastic’ data sets do not. A 2 mm thick water sample produces only about 14% elastic scattering, with the remaining 86% composed of inelastic scattering whose wavelength distribution is approximated by a room-temperature Maxwellian distribution of mean wavelength 〈λ〉 = 1.6 Å (Barker & Mildner, 2015 ▸). The almost purely incoherent scattering from the water should be approximately *q* independent. The small upturn at large *q* is likely to be due to the detector efficiency increasing at large angles, as described by Brûlet *et al.* (2007 ▸), enhanced by the shorter wavelengths produced by the large inelastic contribution. Fig. 6 ▸(*a*) shows that the observed extraneous scattering is significantly increased with the plastic lining, except for *L* = 1.3 m. The latter effect is due to the scattering geometry which, for *L* < 1.4 m, is such that the scattering from the vessel lining cannot reach the detector. Fig. 6 ▸(*b*) shows the ratio of the scattering with and without the plastic lining. The excess background peaks at over 8% of the signal at *L* = 6 m and then decays by 2% as *L* increases. This indicates that the albedo background correction from the detector lining depends upon the detector distance and is therefore not straightforward.

**Figure 7:**
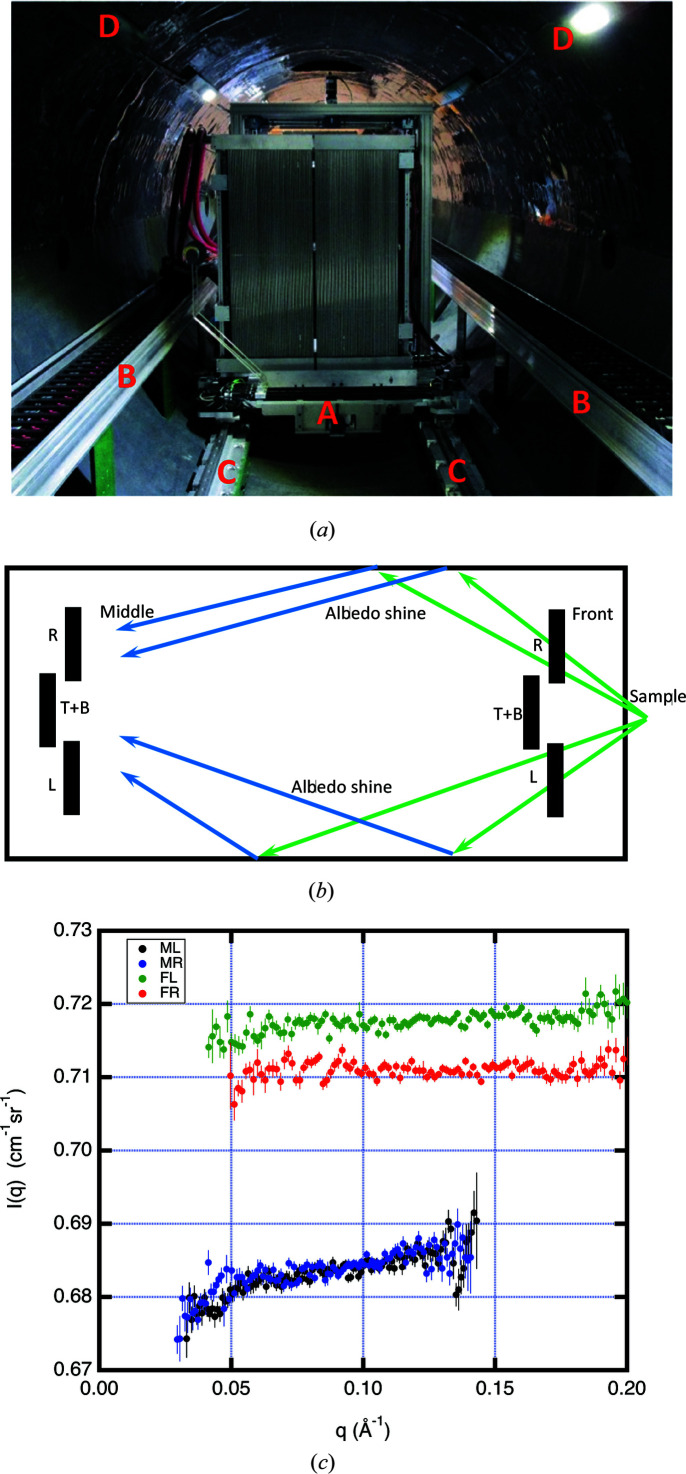
(*a*) A picture of the inside of the vSANS detector vessel. Components that have a potentially high albedo are highlighted. These are (A) the beam-stop support mechanism, wiring and motors, (B) the cable carriers and supports, (C) the metal rails plus supports, and (D) the LED lights plus supports. (*b*) A top-view sketch, showing how the vSANS front carriage blocks a large fraction of the albedo shine from the interior of the detector vessel. The detector panels are labeled L (left), R (right), T (top) and B (bottom). (*c*) A plot of the scattering from a 0.16 cm thick PMMA sample measured on the vSANS instrument. The first measurement used the middle carriage’s left (ML, black) and right (MR, blue) detector panels at *L* = 6 m, with the front carriage at *L* = 0.7 m. In this configuration, the front carriage detectors prevent the majority of sample-scattered neutrons from reaching the vessel lining. The second measurement used the front carriage’s left (FL, green) and right (FR, red) panels at *L* = 4 m, with the middle carriage at *L* = 7 m. In this configuration, the front panels see the full shine from the vessel, causing a 4% increase in the observed scattering.

The vSANS instrument is designed to use three detector carriages to extend the *q* range covered in a single measurement with widely differing sample-to-detector distances *L*. Typically, *L* for the middle carriage is five times *L* for the front carriage, and frequently the measured data have been found not to overlap due to the scattered background appearing preferentially in the front carriage detectors. The D33 instrument at the ILL (Dewhurst *et al.*, 2016 ▸) and the Bilby instrument at ANSTO (Sokolova *et al.*, 2019 ▸) are other examples of SANS instruments where multiple detector carriages are used. Fig. 7 ▸(*a*) shows the interior of the vSANS instrument detector vessel. The top half of the vessel is lined with B-Flex-S and the bottom half between the cable carriers is lined with B-Flex-I. Also shown are a number of unshielded surfaces that may produce enhanced scattering: (A) metal beam-stop support, (B) plastic cable carrier on a metal support, (C) metal rails, and (D) LED lights and supporting metal framing. The combination of higher-albedo interior materials used on the vSANS vessel is expected to increase the average albedo 〈*F*〉, and consequently the backscattered background, by about a factor of five over the NGB 30 m SANS instrument.

Two scattering measurements were made with a *t* = 1.6 mm thick PMMA sample to highlight the data-overlap problem using multiple detector carriages. The front and middle carriages both use four (left, right, top and bottom) detector panels to form an adjustable picture frame. Fig. 7 ▸(*b*) shows schematically the layout with the middle carriage positioned at *L* = 6 m and the front carriage positioned at *L* = 0.7 m used in the first measurement. In this position, the front carriage blocks a large fraction of the large-angle scattering from reaching the vessel lining. In the second measurement the front detector is 4 m from the sample and is unobstructed. Fig. 7 ▸(*c*) shows the scattering from both measurements, which resulted in a 4% increase in the measured *q*-independent scattering for the front carriage with respect to the middle carriage.

In order to mitigate this background, a low-albedo material, such as cadmium sheet, should be used for the vessel lining. If possible, all surfaces in the line of sight of the sample should also be covered in a low-albedo material. This includes the plastic cable carriers, metal rails and other metal framing shown in Fig. 7 ▸(*a*). Alternatively, the albedo shine can be blocked by supporting multiple shielding curtains inside the vessel, oriented perpendicular to the lining surface. The spacing of the curtains is such that they block all lines of sight between the detector and the vessel lining and must not interfere with the movement of the carriages and the cable carriers. Note that the detector side of the curtain needs a low-albedo covering to minimize background from beam-stop scattering. An alternative is to support the screens on the front of the carriage, as is done on the D22 instrument at the ILL.

## 5. Sample-environment albedo background   

The sample can scatter neutrons in all directions into the surrounding sample environment. Large-angle scattering processes include incoherent scattering, inelastic scattering and Bragg scattering. The SANS instrument design typically allows measurement of scattering angles up to 30°, which requires a transparent window such as single-crystal silicon with a diameter as large as 300 mm. The large window allows scattering from a large volume around the sample to be positioned to have line of sight to the detector. Sample-environment components within this volume need to be effectively shielded or designed to minimize the extraneous scattering.

The most commonly used sample environments for SANS are temperature-controlled multiple-position sample-holder blocks. To test for differences in albedo background contributions from blocks of different design, the scattering from a 1 mm thickness of water was made on the vSANS instrument. A broad wavelength range was used, defined by a Be filter combined with a supermirror reflector (Dewhurst, 2012 ▸) and no velocity selector, thus limiting the wavelength range to 4 ≤ λ ≤ 8 Å. The data were corrected for background using 1 mm path-length amorphous silica (‘banjo’) cells for the water sample, an empty cell and, filled with boron carbide powder, dark-current runs. To correct for beam attenuation from windows in the blocks, the beam current was measured independently for each block. The beam current was then used to put the data on an absolute scale *I*(*q*) of cm^−1^ sr^−1^. Only data from the middle carriage ML and MR detectors at *L* = 4 m were used in the calculations. In order to isolate subtle differences in background arising from different sample positions, all three samples, water, empty cell and B_4_C cell, were run in the same sample position in each block.

**Figure 8:**
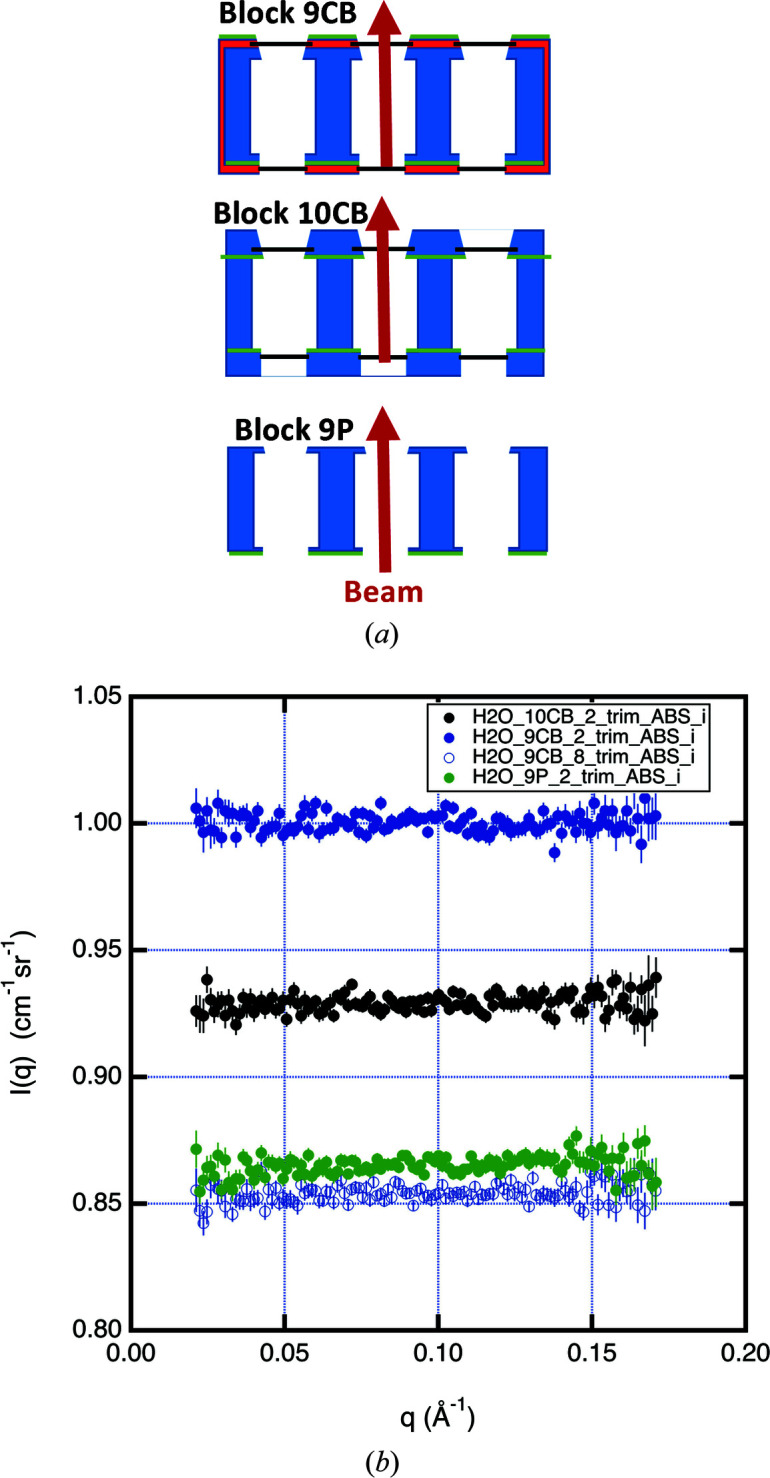
(*a*) A horizontal cut at beam height through the three multiple-position sample blocks used in the Section 5 measurements. Materials in the blocks are color coded as aluminium (blue), amorphous silica (black), plastic (red) and shielding (green). Only block positions 1 to 3 are shown and the neutron beam position 2 in the measurements is indicated by the red arrows. (*b*) A plot of the scattering from a 1 mm thickness of water corrected for empty-cell and beam-blocked backgrounds obtained by inserting the same three cells into the same block position, second from the end, in all three different sample holders at room temperature: 10CB block (black), 9CB (blue) and 9P (green). The open blue circles for the 9CB device were obtained by adding a Cd shielding sheet on the detector side of the block.

The three sample-holder blocks shown schematically in cross section in Fig. 8 ▸(*a*) are commonly used at our facility. The upstream reactor sides of the blocks all contain Cd or ^10^B-Al guard shielding with openings for the beam. The 9P aluminium block has nine rectangular wells for positioning samples and is used under ambient conditions. The 10CB has ten positions and has temperature control using a flow of an ethyl­ene glycol and water mixture through a thermostat bath. Four fluid channels run the length of the block, located a radial distance of 60 mm from the center of the sample. The hydrogen-containing fluid in the channels is the highest-albedo material in this block. The 9CB has nine positions built from three separate aluminium blocks. Each block is temperature controlled via a Peltier device mounted underneath, and excess heat is removed by fluid channels located in the base. Each of the three internal blocks is covered on all sides by plastic sheet. For these measurements, Cd sheet was added to the detector-side exterior of positions 7–9.

Fig. 8 ▸(*b*) shows the difference in the water scattering cross section, *I*(*q*), measured in the second position of all three blocks and in the eighth position of 9CB where shielding was added to the detector side. Without the additional shielding, the 9P, 10CB and 9CB blocks produced scattering enhanced by 1, 7 and 16%, respectively. The enhancement in scattering from 9CB is likely to be caused by scattering in the plastic sheeting surrounding the block, while the 10CB enhancement is probably from the four fluid channels. The much smaller enhancement in 9P is likely to be from the aluminium block. Further measurements were made comparing scattering differences between the end and interior positions of each block. The 9P block showed the smallest difference between positions. Based upon these measurements, the 10 CB block was reassembled with shielding placed on both sides of the sample well. The water measurements were repeated with the reassembled 10CB and 9P blocks, which produced scattering within 1% of each other. The vendor for the 9CB block is also in the process of modifying its design to incorporate shielding on the detector side of the sample. Another method of mitigating sample-environment background is to use a lower-albedo material in the sample-cell construction. Replacing Al with Ti is advantageous due to its higher absorption cross section and good corrosion resistance for liquid-sample holders. BN has also been machined to hold quartz cells.

## 6. Discussion and conclusions   

Albedo scattering affects the accuracy of SANS measurements in a number of ways. The scattering from the beam stop, and rescatter from the vessel lining, enhance the empty beam background. The enhancement is typically only observable when the detector is far from the sample and air and window scattering have been minimized. The enhanced background from the beam stop only adversely impacts the statistical error of weakly scattering samples. The extraneous scattering from the sample environment and detector lining has a more insidious effect because the absolute scattering intensity from incoherent scattering by the sample is actually enhanced. The incoherent cross section is found to be enhanced by the detector vessel lining on the vSANS instrument by 4% and by the 9CB sample block by a further 16% for water samples. Both water and single-crystal vanadium are absolute scattering standard samples that utilize incoherent scattering (Wignall & Bates, 1987 ▸); therefore, a background-enhanced standard sample signal will cause an over-estimation of the scattering cross section of samples. In data analysis, the in­coherent scattering from the sample must be removed from the coherent signal. For dilute biological samples in a dilute salt, partially deuterated, water buffer, the enhanced background can be accurately subtracted by measuring the buffer solution separately (Svergun *et al.*, 2013 ▸). However, in concentrated samples or in experiments where the buffer solution is not measured separately, the background will remain enhanced. In addition, these measurements indicate the enhanced incoherent cross section also depends upon the sample-to-detector distance. For instruments using multiple carriages, such as vSANS at the NCNR, D33 at the ILL and Bilby at ANSTO, the front-carriage detector panels variably screen background affecting the downstream carriage panels, producing differences in the inferred absolute scattering signal.

In order to assess the best-performing materials with respect to their albedo, reflective scattering from 16 commonly used neutron-shielding materials has been measured on an absolute scale at five cold-neutron wavelengths. Many of the polycrystalline materials had scattering dominated by Bragg diffraction at shorter wavelengths. At cold-neutron wavelengths with λ > 2*d*_m_, Bragg diffraction is absent, making inelastic and incoherent scattering the dominant sources. The measured albedos agreed with the observed changes in background obtained from the various choices of beam-stop and detector-lining materials.

To mitigate the background from the detector vessel lining, lower-albedo materials such as cadmium should be used in preference to flexible boron-containing elastomers (B-Flex) which exhibit excessive albedo due to hydrogen scattering. Replacing the current B-Flex with Cd on the NCNR vSANS vessel and covering exposed aluminium in all SANS vessels is expected to lower this background source by up to one order of magnitude. In addition to high absorption with low-energy γ or low γ production, it is also critical that the beam stop has the lowest albedo possible. The best beam-stop materials in this respect are ^6^LiF or ^10^B_4_C. Albedo background from the sample environment is mitigated by placing shielding downstream of the sample, but with openings that allow unimpeded passage of the sample’s SANS to the detector. We have demonstrated that attention to all three of these background sources has measurable benefits for the accuracy of SANS measurements.

## Figures and Tables

**Table 1:** A list of the 16 materials used in this study, plus a material commonly used at other facilities (^10^B_4_C; Dewhurst *et al.*, 2016 ▸), including mass density ρ_m_, composition, maximum *d* spacing *d*_m_ and absorption cross section Σ_abs_ at λ = 1 Å Some materials are proprietary, so composition information is limited. Materials contain natural isotope proportions unless noted otherwise; transmission measurements were used to verify Σ_abs_ in these cases. The thermal neutron cross sections were obtained from Sears (1992 ▸). Material densities and maximum crystalline *d* spacings *d*_m_ were obtained from the *CRC Handbook of Chemistry and Physics* (Weast, 1983 ▸). Some materials are predominantly amorphous (Amor). The alloy designations for the steel and aluminium alloys are 1020 and 6061, respectively.

Material	ρ_m_ (g cm^−3^)	Composition (at.%)	*d*_m_ (Å)	*A* = Σ_abs_/λ (Å^−1^cm^−1^)	Σ_inc_ (cm^−1^)	Σ_coh_ (cm^−1^)	Σ_scat_/*A* (Å)
Al	2.70	Al 98%, Mg 1%, Si 1%	2.338	7.8 × 10^−3^	4.9 × 10^−4^	0.090	12
^10^B-Al	2.70	Al 88%, ^10^B 12%	2.338	17	0.014	0.11	7.3 × 10^−3^
^10^B_4_C	2.34	^10^B 80%, C 20%	6.07	231	0.32	0.023	1.5 × 10^−3^
BN	2.1	B 50%, N 50%	3.309	22	0.11	0.69	0.036
B-Flex	-I	Proprietary	Amor	15			
	-M			17			
	-S			10			
Cd	8.65	Cd 100%	2.81	65	0.16	0.14	4.6 × 10^−3^
Gd	7.9	Gd 100%	3.14	836	4.6	0.34	5.9 × 10^−3^
LDPE	0.92	H 67%, C 33%	Amor	0.015	6.3	1.1 × 10^−3^	420
Li-Flex	1.76	^6^Li 29%, F 29%, H 22%, C 17%, O 3%	Amor	16	1.85	0.032	0.12
^6^Li-glass	2.5	O 45%, Al 21%, ^6^Li 18%, Si 16%	Amor	12	0.019	0.15	0.014
^6^LiF	2.6	^6^Li 50%, F 50%	2.327	33	0.029	0.23	7.8 × 10^−3^
PMMA	1.18	H 53%, C 33%, O 14%	Amor	0.011	4.6	0.013	420
PTFE	2.2	F 67%, C 33%	4.902	3.4 × 10^−4^	6.9 × 10^−5^	0.36	1.1 × 10^3^
SiO_2_	2.2	O 67%, Si 33%	Amor	2.1 × 10^−3^	8.8 × 10^−5^	0.23	110
Steel	7.87	Fe 99%, C 1%	2.027	0.12	0.034	0.95	8.2

**Table 2:** Beam current *I*_B_ measured for different wavelengths The third column shows the count rate in the rear-facing detector array with the Cd beam stop.

λ (Å)	*I*_B_ beam current (s^−1^)	Cd beamstop (s^−1^)
4.5	8.50 × 10^6^	368
6	6.09 × 10^6^	102.8
8	2.79 × 10^6^	80.5
12	4.72 × 10^5^	13.7
15	1.40 × 10^5^	4.93

**Table 3:** The albedo *F* measured for the different shielding materials at neutron wavelengths λ = 4.5, 6, 8, 12 and 15 Å, together with the optical thicknesses τ at each wavelength extreme The optical thicknesses τ were either measured by transmission or obtained by calculation from the material composition for optically thick (τ > 6) samples. The uncertainty in each quantity is within the least significant digit.

		τ		*F*				
Name	*t* (mm)	4.5 Å	15 Å	4.5 Å	6 Å	8 Å	12 Å	15 Å
Al (thin)	1.5	0.016	0.030	9.1 × 10^−3^	8.4 × 10^−4^	8.5 × 10^−4^	1.1 × 10^−3^	1.3 × 10^−3^
Al (thick)	29	0.32	0.59	0.12	0.020	0.020	0.023	0.026
^10^B-Al	2.5	5.8	19.3	4.9 × 10^−4^	1.6 × 10^−4^	1.1 × 10^−4^	1.1 × 10^−4^	1.0 × 10^−4^
BN	6.4	63	211	7.8 × 10^−4^	3.6 × 10^−3^	4.0 × 10^−4^	4.2 × 10^−4^	6.2 × 10^−4^
B-Flex-I	3.3	22	74	0.014	0.012	0.011	9.4 × 10^−3^	8.7 × 10^−3^
B-Flex-M	2.2	16.3	54	6.5 × 10^−3^	5.9 × 10^−3^	5.2 × 10^−3^	5.3 × 10^−3^	5.5 × 10^−3^
B-Flex-S	2.0	9.4	31	0.097	0.12	0.13	0.15	0.16
Cd	0.5	15	49	5.5 × 10^−4^	4.1 × 10^−4^	3.7 × 10^−4^	3.3 × 10^−4^	3.6 × 10^−4^
Gd	0.13	47	157	2.7 × 10^−4^	2.4 × 10^−4^	1.7 × 10^−4^	1.4 × 10^−4^	1.1 × 10^−4^
LDPE	13	6.2	7.2	0.82	0.79	0.70	0.65	0.64
^6^Li-Flex	2.3	17	55	7.1 × 10^−3^	5.9 × 10^−3^	5.0 × 10^−3^	3.8 × 10^−3^	3.2 × 10^−3^
^6^Li-glass	10	54	180	1.2 × 10^−3^	1.0 × 10^−3^	6.7 × 10^−4^	3.4 × 10^−4^	3.0 × 10^−4^
^6^LiF	3.0	46	150	9.2 × 10^−4^	2.7 × 10^−4^	2.3 × 10^−4^	2.3 × 10^−4^	1.7 × 10^−4^
PMMA	6.4	3.0	4.7	0.64	0.62	0.57	0.55	0.54
PTFE	10	0.31	0.16	0.15	0.088	0.36	0.12	0.084
SiO_2_	19	0.36	0.22	0.14	0.19	0.22	0.090	0.075
Steel	22	1.5	5.0	0.045	0.031	0.029	0.028	0.028

**Table 4:** The fraction of the beam *I*_B_ that appears as detector background *k*_m_ with different beam-stop materials and vessel linings on the NGB 30 m SANS instrument The incident beam current was *I*_B_ = 1.8 × 10^6^ s^−1^. A dark-current background was subtracted and the inner 0.25 × 0.25 m area around the beam stop was excluded to remove the effect of the parasitic scattering halo around the beam stop. For comparison, the albedo *F* for each material at λ = 4.5 Å is included in the last column. For the Cd lining, the vessel area coverage was 90%, with the remaining 10% area being the exposed Al rail supports, yielding an average albedo 〈*F*〉 = 0.013 (see Table 5). For the PMMA lining (0.9 mm thick) covering 56% of the area (leaving 34% Cd and 10% Al), the average albedo 〈*F*〉 = 0.11. The addition of PMMA plastic to the lining increased the observed background fraction *k*_m_ by a factor of three to seven, depending upon the beam-stop material.

Beam-stop material	*k*_m_ × 10^6^, Cd lining	*k*_m_ × 10^6^, PMMA lining	*F* at λ = 4.5 Å
Cd	0.6	1.7	5.5 × 10^−4^
^6^Li-Flex	2.9	16.9	7.1 × 10^−3^
B-Flex-I	4.5	33.5	0.014
PMMA+Cd	156	621	0.17

**Table 5:** Calculated average albedo 〈*F*〉 for the NGB 30 m SANS vessel lining with and without the addition of PMMA plastic sheets The fractional surface areas of the linings containing Cd, Al and PMMA are listed in columns 2 and 3. The PMMA sheets are calculated to boost the average albedo 〈*F*〉 by a factor of 8.5 at λ = 4.5 Å. Included in the last column are the albedos *F* for each beam-stop material.

Material	Lining = Cd only	Lining = PMMA + Cd	Material *F* at λ = 4.5 Å
Cd	90%	34%	5.5 × 10^−4^
Al	10%	10%	0.12
PMMA	0%	56%	0.17
Lining 〈*F*〉	0.013	0.11	
